# CSA: An efficient algorithm to improve circular DNA multiple alignment

**DOI:** 10.1186/1471-2105-10-230

**Published:** 2009-07-23

**Authors:** Francisco Fernandes, Luísa Pereira, Ana T Freitas

**Affiliations:** 1 Instituto de Engenharia de Sistemas e Computadores: Investigação e Desenvolvimento (INESC-ID), R. Alves Redol 9, 1000-029 Lisboa, Portugal; 2 IPATIMUP (Instituto de Patologia e Imunologia Molecular da Universidade do Porto), R. Dr. Roberto Frias s/n, 4200-465 Porto, Portugal; 3 Medical Faculty, University of Porto, Al. Prof. Hernâni Monteiro, 4200-319 Porto, Portugal; 4 Instituto Superior Técnico – Universidade Técnica de Lisboa (IST/UTL), Av. Rovisco Pais, 1049-001 Lisboa, Portugal

## Abstract

**Background:**

The comparison of homologous sequences from different species is an essential approach to reconstruct the evolutionary history of species and of the genes they harbour in their genomes. Several complete mitochondrial and nuclear genomes are now available, increasing the importance of using multiple sequence alignment algorithms in comparative genomics. MtDNA has long been used in phylogenetic analysis and errors in the alignments can lead to errors in the interpretation of evolutionary information. Although a large number of multiple sequence alignment algorithms have been proposed to date, they all deal with linear DNA and cannot handle directly circular DNA. Researchers interested in aligning circular DNA sequences must first rotate them to the "right" place using an essentially manual process, before they can use multiple sequence alignment tools.

**Results:**

In this paper we propose an efficient algorithm that identifies the most interesting region to cut circular genomes in order to improve phylogenetic analysis when using standard multiple sequence alignment algorithms. This algorithm identifies the largest chain of non-repeated longest subsequences common to a set of circular mitochondrial DNA sequences. All the sequences are then rotated and made linear for multiple alignment purposes.

To evaluate the effectiveness of this new tool, three different sets of mitochondrial DNA sequences were considered. Other tests considering randomly rotated sequences were also performed. The software package Arlequin was used to evaluate the standard genetic measures of the alignments obtained with and without the use of the CSA algorithm with two well known multiple alignment algorithms, the CLUSTALW and the MAVID tools, and also the visualization tool SinicView.

**Conclusion:**

The results show that a circularization and rotation pre-processing step significantly improves the efficiency of public available multiple sequence alignment algorithms when used in the alignment of circular DNA sequences. The resulting alignments lead to more realistic phylogenetic comparisons between species.

## Background

Genomic sequence alignment tools have been playing an important role in comparative genomics and phylogenetic reconstruction. However, traditional sequence alignment algorithms based on dynamic programming are very inefficient when long genome sequences needed to be aligned. To tackle this problem several heuristic based methods have been proposed. The most popular progressive multiple sequence alignment (MSA) method is ClustalW [1,2], to which access is provided by a number of web portals. Other methods like T-COFFEE [3], DIALIGN [4], MUSCLE [5], MLAGAN [6], MAVID [7], and MAUVE [8] are also widely used. Despite the fact that these tools are heuristic based and sometimes lead to poor biologically plausible alignments, they were also developed to deal only with linear genomic sequences. When applied to circular genomes, the results become extremely sensitive to the exact place where the genomic sequence begins.

This limitation is very important since circular DNA sequence alignments are central to a number of biological problems. Every cell has some kind of genome that is circular. Prokaryotic genomes are circular and many bacteria possess extra circular DNA molecules, the plasmids. Eukaryotic cells also contain organelles which possess circular DNA molecules: the mitochondrial DNA (mtDNA) inside mitochondria in all cells; and chloroplast DNA inside chloroplasts in plant cells.

MtDNA has long been used for phylogenetic analyses. In fact, the absence of recombination in this genome enables an easy and direct inference of the phylogenetic evolution and its fast mutation rate leads to a high discriminative power. Until recently, phylogenetic reconstructions were based on certain regions of the mtDNA molecule, mainly the protein-coding gene cytochrome b when comparing different species [9] and hypervariable regions on D-loop when comparing human populations (e.g. [10]). But the high recent throughput of automatic sequencing techniques is offering the possibility to study complete mtDNA genomes in humans (see revision in [11]) and in other species (ex: *Mus musculus*, [12]). By the end of April 2009 there were around 5,650 human mtDNA complete genomes in GenBank [13] and 1,750 complete mtDNA which should be used as reference sequence for the diverse species in RefSeq [14]. The blind application of standard phylogenetic analyses in these massive datasets without concern to the circularity of these molecules will lead to the overestimation of genetic distances between species.

Sequencing, the technique employed to determine bases constituting the DNA molecule is performed in fragments, generally overlapping in the ends so that an order can be inferred for constructing the map of the molecule. But the place where a circular genome begins is totally irrelevant and arbitrary. For instance, the first team sequencing the human mtDNA [15] decided to begin numbering more or less in the middle of a region designated control region or D-loop; however, the chimpanzee mtDNA sequence has position number one placed in tRNA phenylalanine, which will be position 577 in human mtDNA. Due to this arbitrarily first position definition, a false high genetic distance would be obtained from the alignment between human and its closest species, by using available sequence alignment tools. A total of 576 gaps would be added to the beginning of the chimpanzee sequence and around 563 gaps would be added to the end of the human sequence.

Algorithms for the problem of cyclic sequence alignment have already been proposed in the computer science research field. However, like optimal MSA methods, most existing optimal methods that handle this kind of sequences are very time consuming and seldom used. A simple extension of a general MSA dynamic programming algorithm can be used to compute the edit distance between two cyclic sequences, but requires a quadratic time computation complexity [16,17]. Several other algorithms that explore suboptimal solutions have also been proposed [18,19], reducing the practical execution time. However, these works present experiments that consider only the cyclic use of the Levenshtein metric [17].

Based on algorithms closely related to the ideas described above, two software packages have been recently proposed to align circular DNA genome sequences: the Circal package [20] and the Cyclope package [21]. The algorithm implemented in the Circal package uses a complex gap cost function and can only deal with short sequences, less than a thousand characters, due to its time complexity. The Cyclope package includes an implementation of an exact and a heuristic method with time complexities that are prohibitive if it is used to align several sequences with several thousands of base pairs. For this particular package, the authors claim that it should be used only to obtain a rough first solution of the multiple alignment and that the sequences should be realigned with a standard linear alignment package like ClustalW.

In this paper, we present the CSA tool , a very efficient algorithm that finds the best rotation for a set of circular genome sequences that are to be aligned. Firstly, the genomic sequences are circularized. In a second stage the best rotation is calculated based on the largest chain of non-repeated blocks that belongs to all the sequences. These maximum common blocks are obtained with the help of a *generalized cyclic suffix tree*, which is a new concept introduced in this work. At the end of the process, the users can visualize all the identified common blocks, obtaining a precise idea on how these regions are conserved along the genomic sequences. The new rotated sequences are made available for download and can be submitted to public available MSA tools. At the developed website, several commonly used MSA and visualizations tools are proposed.

## Implementation

The purpose of the proposed algorithm is to find the best rotation among all the possible rotations of each circular genome sequence, in order to improve subsequent multiple sequence genome alignment. Unlike previous algorithms that pursued the same goal, the proposed algorithm is, to the best of our knowledge, the first one that is able to do this task in linear time. This was accomplished by employing the highly efficient suffix tree data structure [22].

### Suffix Trees

In general terms, a suffix tree for a given string is an advanced data structure shaped like an upside down tree that stores all the suffixes of the string and that can be used to efficiently solve many complex string problems. An in-depth explanation of this data structure is outside the scope of this article, but a detailed overview of suffix trees including construction methods and applications can be found in a number of sources (e.g. [23]). In this work we follow many of the definitions presented in that reference. In particular, strings will be denoted as sequences, which correspond to DNA sequences.

### Cyclic Suffix Trees

To be able to represent all the rotations of a cyclic sequence S of length n, we introduce the concept of *cyclic suffix tree*. A cyclic suffix tree is a suffix-tree-like structure which represents all the rotations of the sequence (instead of all its suffixes, as is normal for suffix trees). The construction algorithm follows Ukkonen's suffix tree construction method [24] but with some subtle modifications, namely in the implementation of *suffix links *and *open leaves *(see [23] for a detailed description of these concepts). In our case, suffix links at leaves are treated as connecting successive rotations instead of successive suffixes. The role of the open leaves is also changed so that the resulting path label from the root to the end of the leaf has always the same length, n. In this way, if a new leaf for the character at position i is created at depth d, the right pointer of that leaf will be i + (n - d - 1). The result is that all the leaves are at the same depth in the tree, which corresponds to the size of the original sequence. When accessing characters from the edge labels, if a pointer indicates a position k that reaches beyond the end of the original sequence (i.e., k > n), then we must subtract from that position the size of the sequence (i.e., k = k - n).

### The construction algorithm

Based on the previous definitions, the construction algorithm proceeds as originally proposed in [24], producing all the n rotations of the sequence in linear time with no additional effort. In some cases, as a final step, we still need to perform an additional pass through S. Take for example the sequence AAABA. The construction algorithm, as proposed in [24], would stop at the internal node with path label A, and would not report the rotation AAAAB. So, we need to match again all the characters from the beginning of the sequence until the (n-1)-th position to create the last missing rotation. This can easily be done by constructing the cyclic suffix tree of the sequence concatenated with itself (i.e., SS). Using this technique, the algorithm runs in time proportional to 2 n, and we obtain a linear time complexity.

**Figure 1:**
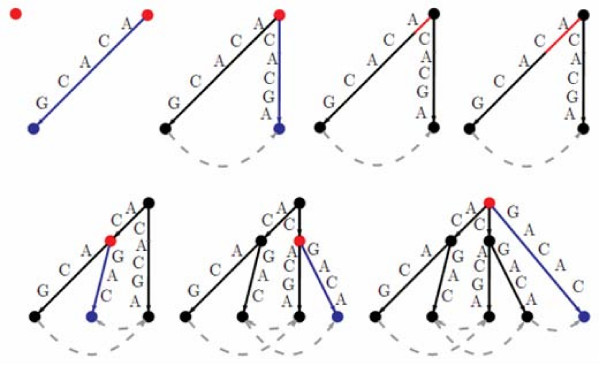
**Cyclic suffix tree step by step construction for the sequence ACACG**.

**Figure 2:**
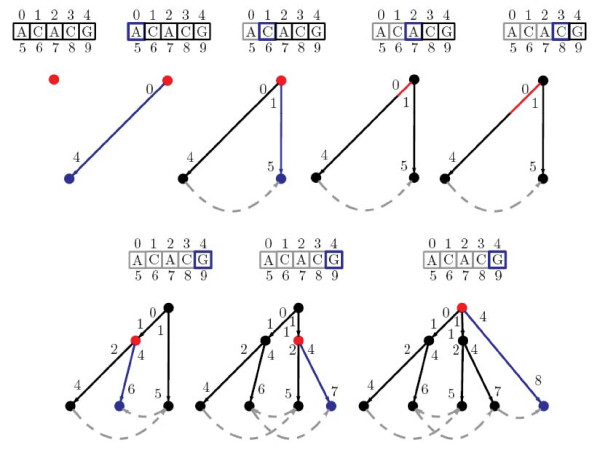
**Cyclic suffix tree step by step construction for the sequence ACACG, showing internal node pointers instead of node labels**.

The example presented in Figure 1 shows all the steps for the construction of the cyclic suffix tree for the sequence ACACG. The current node/position is marked in red and the newly created nodes/edges are coloured in blue. For the sake of simplicity, only the suffix links connecting leaves are shown. Figure 2 presents the same construction but gives a deeper look at the node pointers, showing some of the implementation details of the algorithm.

### Generalized Cyclic Suffix Trees

We have so far presented the algorithm to build a cyclic suffix tree for a single sequence, in linear time. However what we want is to efficiently obtain the best rotation for an entire set of cyclic sequences. For this we build a tree called a *generalized cyclic suffix tree*. A generalized cyclic suffix tree is a tree that stores all the rotations of a set of sequences. In this representation, a node can belong to several different sequences at the same time. Each node in the tree is marked, using a bit vector, with the identifiers of all the sequences that contain that node. A linear time algorithm for the construction of a generalized suffix tree for a set of sequences can be found in [23]. The implementation used in this work is a generalization to deal with cyclic sequences.

**Figure 3:**
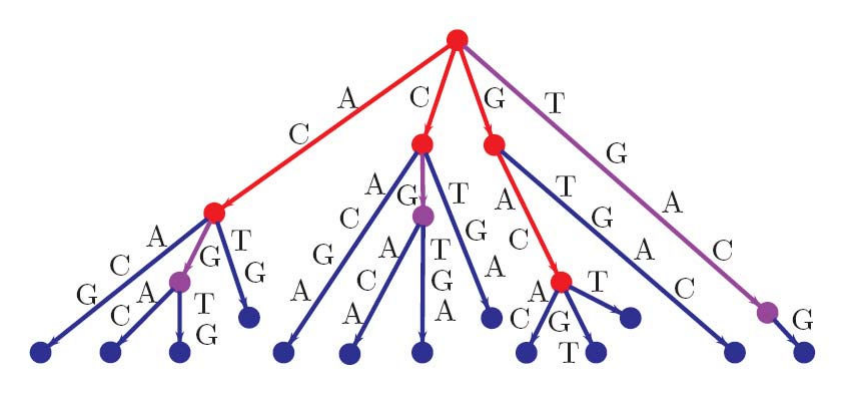
**Example of a generalized cyclic suffix tree for sequences ACACG, CGTGA and TGAC**.

Figure 3 shows a simple example of a generalized cyclic suffix tree for the following set of three sequences: ACACG, CGTGA and TGAC. As one can see, every rotation of every sequence is present in the represented tree. To allow a more clear view, the nodes/edges that belong to all the sequences are marked in red, the ones that belong to only two of the sequences in purple, and the ones corresponding to a single sequence in blue.

### Finding the best rotation

The general idea to obtain the best rotation for each sequence based on all the others is to find the largest chain of longest common subsequences that belongs to all the sequences and then use the position of that highly conserved block chain in each sequence to establish the cutting point. We start by retrieving all the nodes that belong simultaneously to all the sequences. The tree nodes carry a bit vector with this information, so we only need to perform a depth-first search on the tree starting at the root and count the number of sequences in each node. When the sequence count, in a node, falls bellow the total number of sequences, we don't need to search the children of that node because their count will only be lower or equal to the count of the father. Using the suffix links of those nodes, it is now possible to discard all the nodes whose path label corresponds to a suffix of the path label of another node. The result is the set of all maximal blocks common to all the sequences.

To improve the results three other steps were included. One step removes the nodes that appear more than once in at least one of the sequences. This is important because a repeated subsequence that appears in multiple positions inside a sequence could lead to wrong alignments. At this stage, we are left with all the unique maximal common blocks to all the sequences. Next, the second step groups together the blocks that appear consecutively and in the same order in all the sequences. The third and last step consists of taking the largest chain of these blocks and set its start position on each circular sequence as the start position of the new linear sequence.

For the identification of close related regions the minimum block size is not limited. However, since all the suffixes of common subsequences are automatically excluded when the suffix tree is analysed, it is usual that the longest common subsequences have size not inferior to 5 or 6 nucleotides. The only restriction that was included limits the maximum distance between two consecutive blocks in a chain to 10 nucleotides. Since the algorithm purpose is to identify the best rotation for all the sequences based on a similar region, we tried, with this distance restriction, to avoid that other more complex biological events, like gene inversions, deletions or insertions could play a role at this stage. These events will be detected at the final multiple alignment. The length of the chain, or common region, is the sum of the lengths of each one of the intervening blocks.

Consider the example represented in Figure 3 where sequences ACACG, CGTGA and TGAC correspond to a linearization of three DNA cyclic sequences. There are three common blocks to all these sequences: GAC, AC and C. Since AC and C are both suffixes of GAC, they are removed. The remaining block does not occur more than once in the same sequence, so repetitions are not observed. At the end, the single sequence GAC is reported as the largest block chain, leading to the following rotated and linear set of sequences: GACAC, GACGT and GACT.

**Figure 4:**
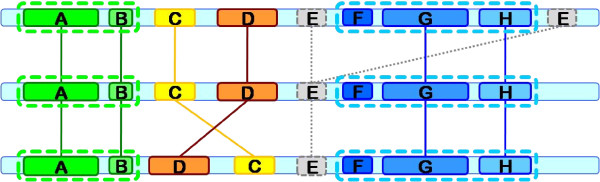
**Example of 4 block chains (A+B, C, D and F+G+H) derived from a set of longest common subsequences, from 3 sequences**.

Figure 4 shows a more elaborated example of the block chains obtained from a set of longest common subsequences present in 3 sequences. All the blocks are shown almost aligned for a better understanding of the concept of block chains, but the sequences can have any random rotation. Block E is automatically discarded because it appears twice in the first sequence. Blocks A and B belong to the same chain since they appear in the same order in every sequence. Blocks F, G and H also form another chain. Blocks C and D are excluded from those chains because they do not have the same order in all the sequences. However, each one of these two blocks forms itself an elementary chain containing a single block. The longest chain corresponds to blocks F, G and H and therefore all the final rotated sequences will start with this chain of blocks.

### Multiple Sequence Alignment

After finding the largest chain of unique common blocks belonging to all the sequences that were circularized, they are again made linear by cutting these sequences at the starting position of the first block from that common chain. The multiple alignment itself can then be easily performed by any linear multiple alignment algorithm. At the CSA tool web site several multiple alignment methods and visualization tools are suggested for further sequence analysis.

### The CSA tool interfaces

**Figure 5:**
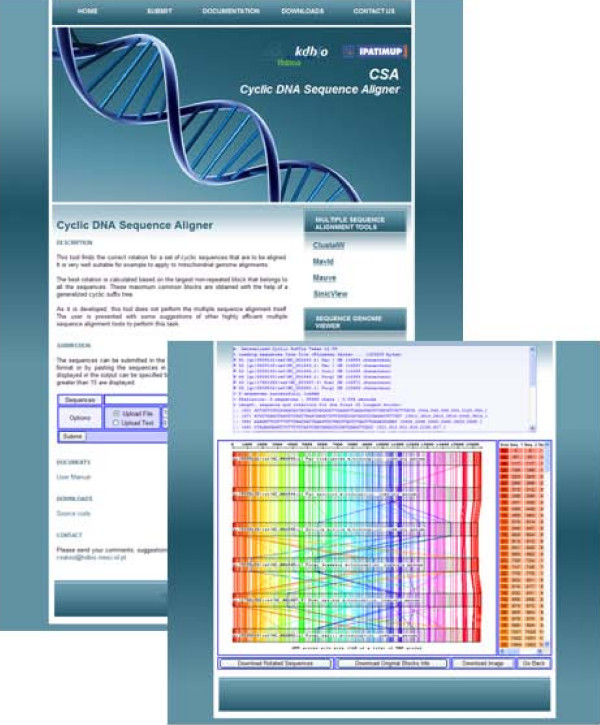
**CSA tool, input and output interfaces**.

The CSA tool is available through a friendly and easy to use web interface. Figure 5 presents the tool's main page and the output results after pre-processing a set of mtDNA Primate sequences.

The genomic sequences can be submitted in the Multi-FASTA format by uploading a file or by pasting the sequences in a text window. The size of the chain of blocks displayed in the output can be specified by the user. By default chains with size higher than fifteen bases are selected and displayed. The minimum size allowed for a chain with only one block is eight since finding conserved blocks of this size in genomic sequences with several thousand base pairs is still statistically significant.

The output page is divided in three main areas where data are displayed. At the beginning the user can find tool execution statistics, including the size and description of each input sequence, the algorithm running time, information about the 20 first longest blocks, and eventually any processing errors. After this general information, the alignment map of the blocks in all the sequences after their optimal rotation is presented, together with a table with all the blocks lengths and positions, following the same colour schema as the alignment map. The colour of the blocks in the results page is not related to their length. The blocks are coloured in a rainbow-like fashion by their positions relative to the first (top-most) sequence. This position-based colouring is more interesting than a length based colouring because it allows, in a much more easy way, the detection of block transpositions among sequences. Before clicking on the alignment image, the table on the right shows the blocks sorted by their length. By left-clicking over a specific section of one of the sequences in the alignment map, the positions table automatically sorts its rows to reflect the correct order of the blocks inside that sequence and it automatically scrolls itself to show the information of the blocks from the selected region/sequence. All the previous described data are available for download at the end of the page.

At the CSA tool web page the user can also find a user manual, the algorithm source code and some example sequences.

## Results and discussion

In order to evaluate the efficiency of the proposed tool and the adequacy to perform the circularization and rotation prior to the alignment, we conducted tests in three sets of mtDNA sequences. The first set includes sequences of 16 Primates, the second set includes sequences of 12 Mammals and the last set is a set of distantly related sequences including 19 mtDNA sequences (the 16 Primates, the *Drosophila melanogaster*, the *Gallus gallus *and the *Crocodylus niloticus*) (Table 1). These datasets are available, as supplementary material, at the tool's web site. Multiple sequence alignments with and without CSA pre-processing were performed using two well known MSA tools: ClustalW [1] and MAVID [7]. Alignment quality was compared in two ways: (1) by evaluating genetic standard measures in the software Arlequin [25]; (2) by using the tool SinicView [26], a visualization environment for comparison of multiple nucleotide sequence alignment tools.

**Table 1:** Species, GenBank accession numbers and mtDNA genome size (bp) used for the tests.

First set (Primates)			Second set (Mammals)		
Species	Accession Number	Genome size (bp)	Species	Accession Number	Genome size (bp)

Pan troglodytes	NC_001643	16554	Artibeus jamaicensis	NC_002009	16651

Pan paniscus	NC_001644	16563	Episoriculus fumidus	NC_003040	17488

Pongo pygmaeus	NC_001646	16389	Ornithorhynchus anatinus	NC_000891	17019

Homo sapiens	NC_001807	16571	Bos taurus	NC_001567	16338

Papio hamadryas	NC_001992	16521	Mus musculus	NC_001569	16295

Hylobates lar	NC_002082	16472	Balaenoptera musculus	NC_001601	16402

Pongo abelii	NC_002083	16499	Equus caballus	NC_001640	16660

Cebus albifrons	NC_002763	16554	Macropus robustus	NC_001794	16896

Nycticebus coucang	NC_002765	16764	Homo sapiens	NC_001807	16571

Tarsius bancanus	NC_002811	16927	Canis lupus familiaris	NC_002008	16727

Lemur catta	NC_004025	17036	Macroscelides proboscideus	NC_004026	16641

Macaca mulatta	NC_005943	16564	Lepus europaeus	NC_004028	17734

Trachypithecus obscurus	NC_006900	16560			

Presbytis melalophos	NC_008217	16543			

Chlorocebus tantalus	NC_009748	16368			

Gorilla gorilla	NC_011120	16412			


Third set (Distantly related sequences)					
			
Species	Accession Number	Genome size (bp)			
			
16 Primates sequences (First set)	----	-----			
			
Drosophila melanogaster	NC_001709	19517			
			
Gallus gallus	NC_001323	16775			
			
Crocodylus niloticus	NC_008142	16830			

In order to get a sense of the statistical significance of the CSA improvement relative to the random situation, tests with 50 sets of control sequences with random cuts (using the third set described) were also conducted. For these sets, the alignment scores and the consensus length obtained when using the ClustalW tool with and without the CSA algorithm were compared.

We also tried to compare the results obtained with the CSA tool followed by a linear DNA multiple sequence aligner, with the results obtained by the algorithms available at the Cyclope package [21]. However, this comparison could not be performed because this software package is not able to deal with sequences with a few thousand bases pairs as the mtDNA sequences. The recommendation obtained by the tool's authors was to perform a manual rotation based on the genes positions and then use a linear DNA multiple sequence alignment algorithm.

### The circularization and rotation in CSA

**Figure 6:**
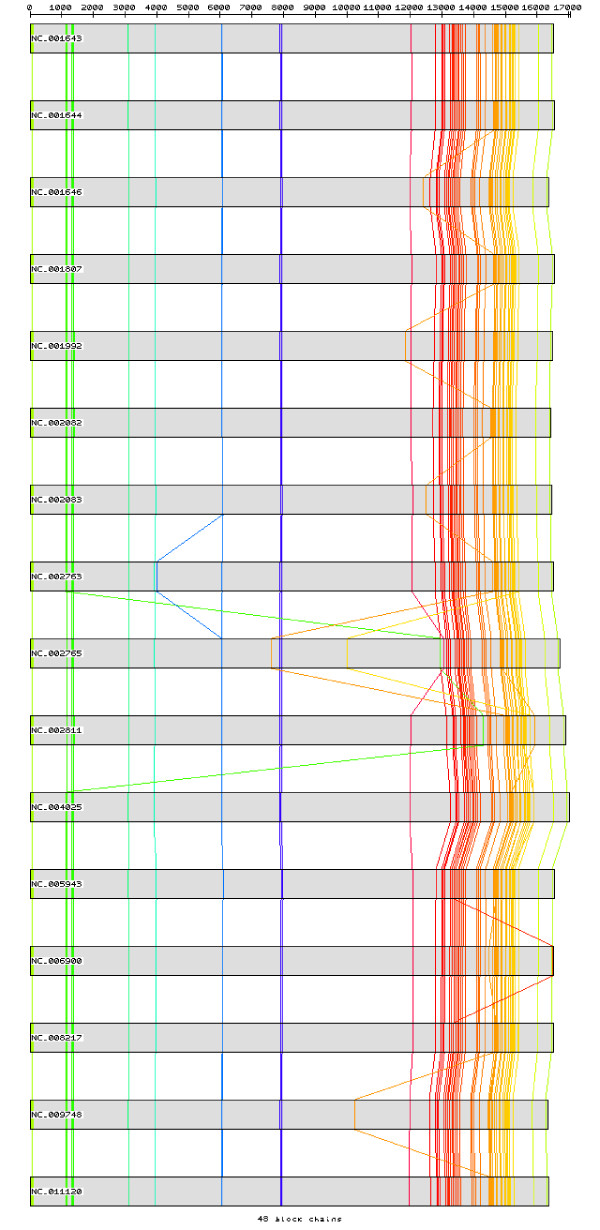
**The 17 blocks with size 15 bp or bigger in Primate mtDNA sequences, after pre-processing with CSA**.

**Figure 7:**
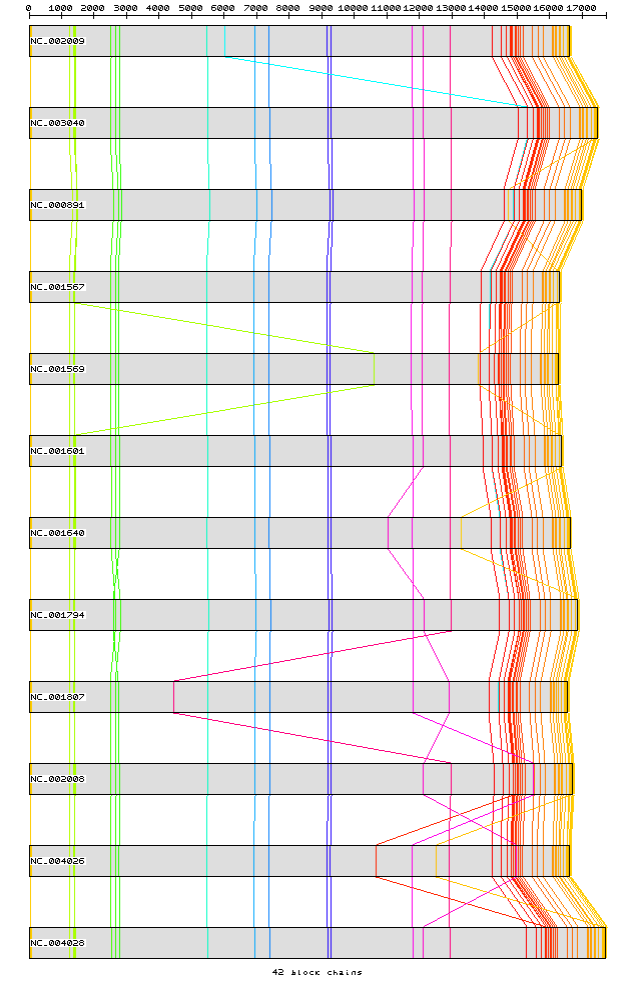
**The 15 blocks with size 15 bp or bigger in Mammal mtDNA sequences, after pre-processing with CSA**.

**Figure 8:**
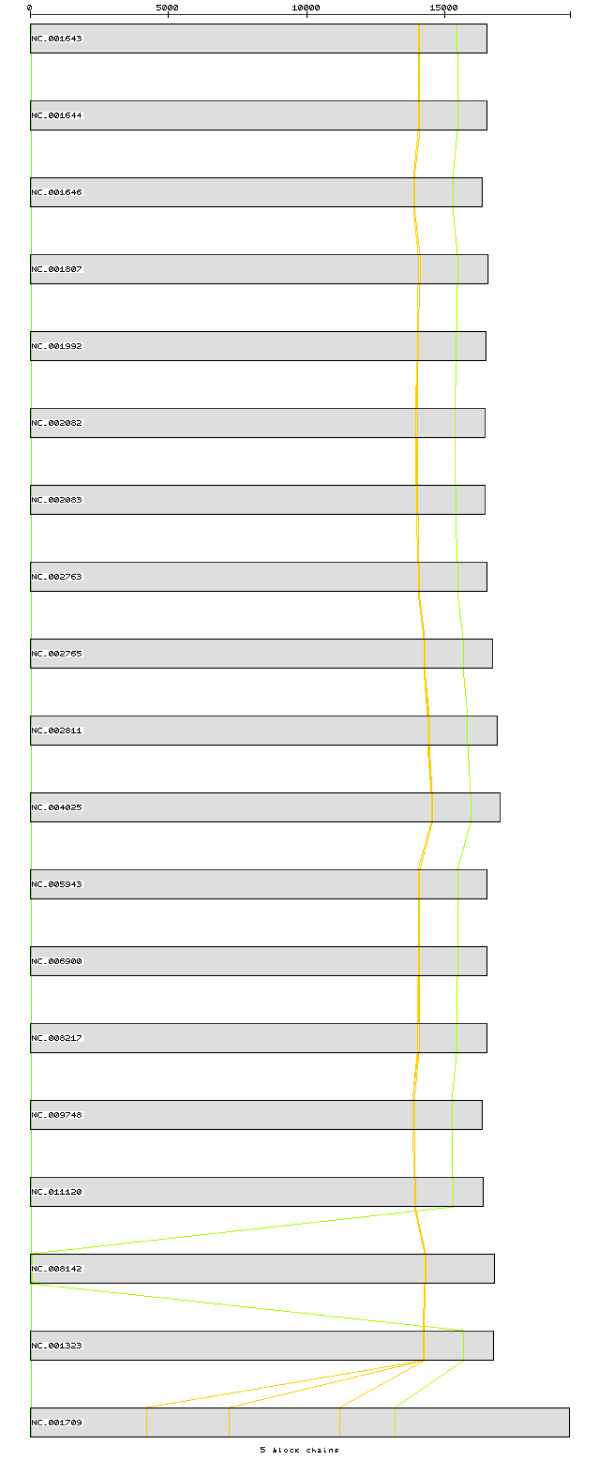
**The 5 blocks with size 8 bp or bigger in the third set of mtDNA sequences, after pre-processing with CSA**.

After pre-processing the set of Primate sequences with CSA, 58 homologous chains of blocks with size 8 or bigger were observed, with the biggest one consisting in a block of 16 bp followed by a gap of one base, a second block of 29 bp followed by a gap of 9 bp and a third block of 15 bp, which matches perfectly the tRNA-Met (Figure 6). Those homologous chains of blocks are shortened when enlarging the analysis to the Mammal set. A total of 51 homologous chains of blocks with size 8 or bigger were still observed, being the biggest one constituted by a block of 25 bp followed by a gap of one base and another block of 21 bp, located at the gene 16 s rRNA (Figure 7). The third set, including the 16 Primates and the outgroup species, reduced the homologous chains of blocks to four unique blocks with size 8 or bigger, the biggest one being a unique block of 10 bp located in the tRNA-Trp (Figure 8). As expected, the bigger homologous chains of blocks are located in regions where the secondary structure must be maintained as it is essential for functionality. Protein-coding genes do not have such high mutation-constraint as the rRNA and tRNA genes due to the property of redundancy of the genetic code: most aminoacids are coded by several codons, the third-codon position being quite irrelevant.

Note, however, that when more distantly related sequences are considered, the common blocks correspond to single blocks of small sizes (more or less 8 bp) that are still statistical significant if mitochondrial DNA is being considered but not true for DNA sequences of bacterial genomes with million base pairs. In this way, additional care must be taken in the analysis of the sizes of the identified common blocks, when using this algorithmic approach with larger circular genomes from distantly related organisms. In these cases we are dealing with the multiple sequence alignment problem of distantly related genomes that is still an open problem.

### Comparing alignment results between alignment tools and with or without CSA

Some biological properties can help in the evaluation of the alignment results: (1) unique deletion of multiple bases instead of multiple deletions interspersing individual nucleotides; (2) higher ratio transition/transversion, as substitutions between the same type of bases are commonest than between different types; (2) the triplet-constraint in protein-coding genes making it more probable to have deletion in multiples of three than other.

As can be observed in Table 2, the genetic diversity standard measures showed lower levels of diversity (mainly the mean number of pairwise differences between sequences) when circularizing and rotating the mtDNA molecules in CSA prior to the alignment, in both alignment tools tested. This fact is stronger in the set of Primates with outgroups than in Mammals and still than in Primates, as phylogenetic distance between compared species decreases. The main difference between circularization and rotation or not of the mtDNA molecules with CSA is in the number of insertion/deletions (indels), which reduces about 1,000 events when applying this pre-processing step.

**Table 2:** Genetic diversity standard measures for Primates, Mammals and Primates with more distantly related sequences, aligned with several alignment tools without and after circularization an rotation in CSA.

		ClustalW	CSA + ClustalW	MAVID	CSA + MAVID
First set (Primates)	size (bp)	18033	17447	18388	17800
	
	polymorphic sites	10910	10295	11290	10673
	
	transitions	9429	9387	9132	9112
	
	transversions	5226	5167	5105	5106
	
	substitutions	14655	14554	14237	14218
	
	indels	2544	1620	3227	2508
	
	Mean no. of pairwise differences	4303 +/- 1939	4084 +/- 1840	4391 +/- 1978	4223 +/- 1903
	
	Nucleotide diversity	0.239 +/- 0.120	0.234 +/- 0.118	0.239 +/- 0.121	0.237 +/- 0.120

Second set (Mammals)	size (bp)	19220	18612	21745	20820
	
	polymorphic sites	12591	11995	15177	14204
	
	transitions	9987	10090	8977	9177
	
	transversions	6625	6607	6195	6336
	
	substitutions	16612	16697	15172	15513
	
	indels	3916	2988	7420	6283
	
	Mean no. of pairwise differences	5640 +/- 2592	5386 +/- 2475	6200 +/- 2849	6044 +/- 2778
	
	Nucleotide diversity	0.293 +/- 0.152	0.289 +/- 0.150	0.285 +/- 0.148	0.290 +/- 0.150

Third set (Primates + Drosophila melanogaster + Gallus gallus + Crocodylus niloticus)	size (bp)	19964	20196	29363	27978
	
	polymorphic sites	17514	17072	26733	25056
	
	transitions	13594	13252	7177	7562
	
	transversions	9777	9328	5238	5453
	
	substitutions	23371	22580	12415	13015
	
	indels	5473	5024	21689	19146
	
	Mean no. of pairwise differences	5892 +/- 2631	5568 +/- 2486	7018 +/- 3133	6729 +/- 3004
	
	Nucleotide diversity	0.295 +/- 0.147	0.276 +/- 0.138	0.239 +/- 0.119	0.241 +/- 0.120

When comparing different alignment tools, ClustalW adds considerable lower amounts of indels than MAVID. A probable cause for this is that MAVID was especially designed for the alignment of large genomes, while ClustalW is much more conservative and takes longer to achieve results.

**Figure 9:**
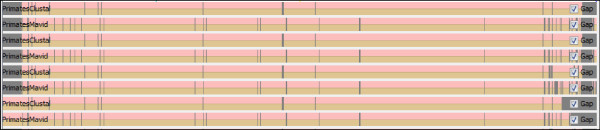
**SinicView comparison between ClustalW and MAVID alignments prior to CSA pre-processing, in four Primates **(NC_001643, NC_001644, NC_011120**and **NC_001807).

**Figure 10:**
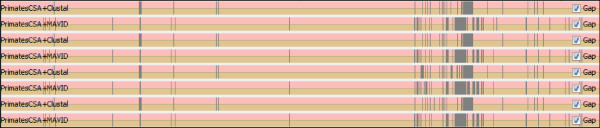
**SinicView comparison between ClustalW and MAVID alignments after circularization and rotation in CSA**.

We tried to investigate where indels were being introduced. This can be visually checked in the tool SinicView, as displayed in Figures 9 and 10. Prior to the CSA application (Figure 9), huge gaps are included in the original tips, which coincide with the most heterogeneous mtDNA region, the control region. After the circularization and rotation (Figure 10), gaps are mainly included in the control region, and a big difference can be found between the alignment tools. Outside the control region, the biggest gap is inserted in a small fragment which is non-coding, being placed between gene COX2 and tRNA-Lys. All the alignment tools allow for non-multiples of three in protein-coding genes to be included, and, for instance, when counting deletions inserted in human protein-coding genes for the Primate set, the ratio of multiple/non-multiple of three deletions is higher in ClustalW than in MAVID (1.2 and 0.5 respectively). MAVID is the less restrictive in having multiple deletions interspersing individual nucleotides.

### Comparing alignment results in the 50 sets of control sequences with random cuts

**Figure 11:**
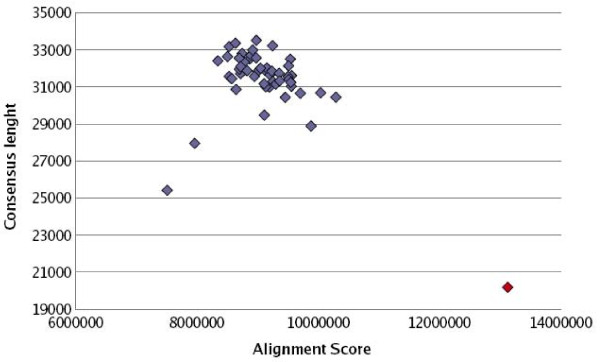
**Plotted results of the 50 test sets of randomly rotated mtDNA sequences (ClustalW in blue and CSA+ClustalW in red)**.

When the third set was used so that each sequence was cut each time in different random positions, resulting in 50 test sets of 19 sequences each, the efficiency improvement of CSA was also evident. It was shown that when using the CSA tool on these sets, they all resulted in the same alignment and its score greatly outperformed the scores of each not CSA-processed test set. Figure 11 presents in blue the consensus length, in bp, versus the alignment score for the 50 test sets when using the ClustalW tool for the multiple sequence alignment. When using the CSA tool as a pre-processing step the alignment score is always the same and the length of the consensus sequence is much smaller (for more details see the supplementary material in the webpage).

## Conclusion

We have demonstrated that the essential step of circularizing and rotating the mtDNA molecules prior to its alignment can significantly improve the efficiency of current multiple sequence alignment tools, developed for the alignment of linear DNA molecules. This pre-processing step leads to more accurate phylogenetic comparisons between species.

To the best of our knowledge the CSA tool is the only web based tool that obtains the best rotation of a set of circular DNA sequences in a very efficient way. The new rotated sequences are made available for further processing and a picture of all conserved block for all the sequences can be found at the result page and can be viewed as a first draft of a future multiple sequence alignment.

Future developments of alignment tools should include more real biological mutation constraints, enabling the use of different assumptions in the different parts of the molecules. It is clear that as sequencing strategies advance further, more information will be obtained for complete genomes, which have necessarily a diverse composition. This is clearly the case of the circular molecules of mtDNA and bacterial genomes being rapidly characterized. For instance, non-coding regions could have a less restrictive rate of gap opening; protein-coding genes should incorporate the rule of multiple of three gaps and be less restrictive for substitution at the third-codon position; third-dimension structure can give additional information for the rRNA and tRNA genes alignment.

## Availability and requirements

**Project name**: CSA: Cyclic DNA Sequence Aligner

**Project home page**: 

**Operating system(s)**: Platform independent

**Programming language**: C

**Other requirements**: none

**License**: None

**Any restrictions to use by non-academics**: Free downloads and usage for academics only

## Authors' contributions

LP and ATF specified the problem and supervised the system implementation. FF implemented the algorithm and coded the application and the web interface. All authors contributed for the writing, read and approved the final manuscript.
